# Outcome Prediction by Combining Initial Clinical Severity With Corticospinal Tract Lesion Load in Patients With Intracerebral Hemorrhage

**DOI:** 10.7759/cureus.82430

**Published:** 2025-04-17

**Authors:** Toshiki Yasukawa, Yuki Uchiyama, Tetsuo Koyama, Kazuhisa Domen

**Affiliations:** 1 Department of Rehabilitation Medicine, Hyogo College of Medicine, Nishinomiya, JPN; 2 Department of Rehabilitation Medicine, Nishinomiya Kyoritsu Neurosurgical Hospital, Hyogo, JPN

**Keywords:** imaging, manifestation, prognosis, recovery, symptom

## Abstract

Objective: This study aimed to assess the predictive accuracy of motor outcomes in patients with intracerebral hemorrhage by integrating the initial severity of hemiparesis and the corticospinal tract lesion load (CST-LL).

Materials and methods: A retrospective analysis was conducted on patients diagnosed with putaminal and/or thalamic hemorrhage who underwent computed tomography (CT) shortly after stroke onset. The CT images were aligned with a standardized brain template to calculate CST-LL. The initial severity of hemiparesis was evaluated using the summed Brunnstrom Recovery Stage (BRS total; range: 3-18). Motor outcomes at the time of discharge from a rehabilitation facility were assessed using the motor component total score of the Stroke Impairment Assessment Set (SIAS-motor total; range: 0-25). A multivariate regression analysis was performed with BRS total and CST-LL as independent variables and SIAS-motor total as the dependent variable.

Results: A total of 61 patients were included in the analysis. The median CST-LL was 1.974 mL (interquartile range (IQR): 1.113-3.311 mL), the median BRS total was 8 (IQR: 4-13), and the median SIAS-motor total was 20 (IQR: 9.5-24.5). Both BRS total and CST-LL were found to be significant predictors of motor outcomes. The estimated t-values were 4.79 for BRS total and −3.29 for CST-LL, indicating comparable contributions of both factors. The developed regression model explained 60.4% of the variance in SIAS-motor outcomes.

Conclusions: The combination of initial clinical severity and CST-LL enhances the predictive accuracy of motor recovery in patients with intracerebral hemorrhage.

## Introduction

Predicting patient outcomes is crucial for planning effective rehabilitation strategies for individuals who have experienced a stroke [1]. Among the various factors influencing recovery, the severity of clinical symptoms in the early stages is particularly important, as it provides critical insights into stroke impact and guides therapeutic and rehabilitative interventions [2]. Therefore, assessing clinical manifestations during the acute phase is essential for predicting outcomes and determining appropriate management strategies in stroke rehabilitation.

Beyond initial clinical severity, the integrity of the corticospinal tract (CST) has been identified as a key factor in predicting functional outcomes, particularly motor recovery in the extremities [3,4]. Studies utilizing magnetic resonance imaging (MRI) have demonstrated that the overlap between the stroke lesion and the CST, known as CST lesion load (CST-LL), correlates with motor function outcomes [5,6]. In addition, computed tomography (CT), which is commonly used in stroke management, especially for hemorrhagic stroke, has been investigated as a potential tool for estimating CST-LL. Some reports suggest that CST-LL derived from CT imaging may aid in outcome prediction, particularly in cases involving putaminal or thalamic hemorrhage [7,8].

Various predictive models incorporating techniques such as machine learning and functional MRI have been developed to estimate stroke outcomes [9-11]. However, these methods often require significant computational resources and prolonged processing times, limiting their feasibility for routine clinical use. In contrast, integrating initial clinical severity assessment with CST-LL derived from standard CT scans offers a practical approach that can be readily implemented in daily clinical practice [8,12]. This study aims to evaluate the clinical utility of combining early clinical severity and CST-LL in predicting outcomes for patients with intracerebral hemorrhage.

## Materials and methods

Patient selection

This retrospective study builds on our previous research investigating the clinical applicability of conventional CT imaging in stroke rehabilitation [8,12]. It included patients diagnosed with intracerebral hemorrhage who were admitted to Nishinomiya Kyoritsu Neurosurgical Hospital between April 2019 and March 2024. Upon admission, patients typically received conservative treatment, such as antihypertensive therapy, with surgical evacuation of hematomas performed when indicated [13]. Rehabilitation interventions, including physical, occupational, and speech therapy, were provided for up to 180 minutes daily, following the Japanese stroke management guidelines [13].

To minimize variability due to pre-stroke health status and lesion location, the study focused on individuals experiencing their first-ever stroke with hemorrhagic lesions in the putamen and/or thalamus who had been functionally independent in daily activities, including unassisted ambulation, prior to the stroke. Patients were excluded if they had a history of neurological disorders such as Parkinson’s or Alzheimer’s disease, exhibited progressive neurological deficits, or had significant comorbidities [12]. To ensure a consistent rehabilitation protocol, only patients subsequently transferred to Nishinomiya Kyoritsu Rehabilitation Hospital, an affiliated long-term rehabilitation facility, were included [12]. The study protocol was approved by the Ethics Committee of Hyogo College of Medicine (approval no. 4666), and informed consent was obtained using an opt-out approach.

CT imaging and processing

CT acquisition and image processing followed previously published methods by our research team [8,12]. Briefly, patients underwent a head CT scan upon admission when stroke was suspected. Imaging was performed using a 320-row detector CT scanner (Aquilion ONE, Canon Medical Systems Corp., Tochigi, Japan) with a standard helical acquisition protocol. Parameters included a tube voltage of 120 kVp, a current of 300 mAs, an in-plane resolution of 0.43 × 0.43 mm (512 × 512 matrix), and a slice thickness of 5 mm.

CT image processing was conducted using FMRIB Software Library (FSL) version 6.0.6 (FMRIB, Oxford, UK) for brain image analysis [14]. Images were thresholded within the Hounsfield unit (HU) range of 0-100 and spatially normalized to a standard CT template [7,8,12]. Spatial normalization accuracy was confirmed by visual inspection. A rectangular volume of interest (VOI) was defined for each patient, encompassing the hematoma in the x, y, and z planes. A lesion mask was created by extracting and binarizing voxels within the VOI that exceeded 50% of the HU threshold [8,12].

**Figure 1:**
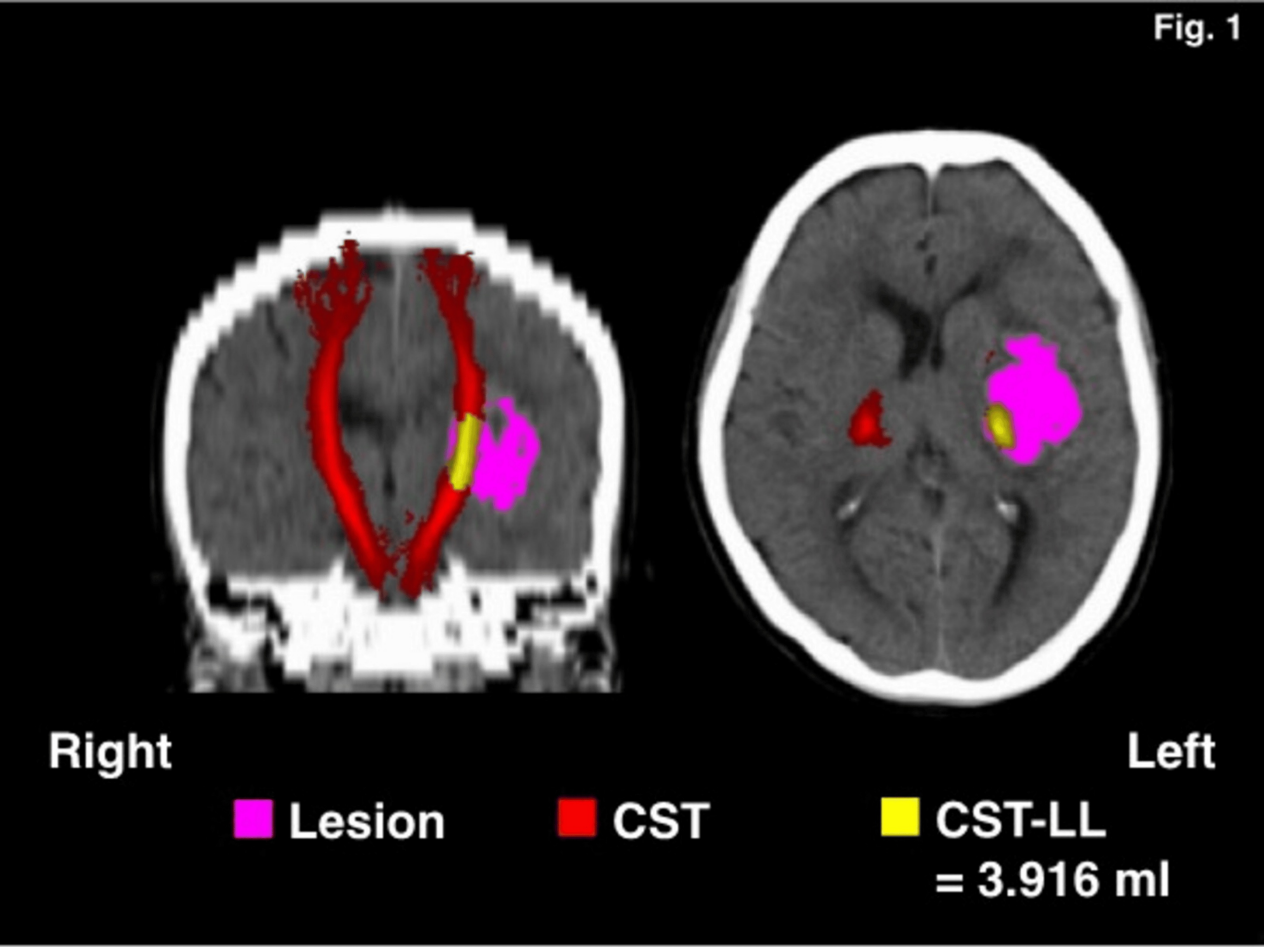
Representative computed tomography images of the brain after spatial registration to a standard brain. CST, corticospinal tract; LL, lesion load.

The CSTs in both hemispheres were delineated using the JHU-ICBM-tracts-maxprob-thr0-1mm.nii.gz template from the FMRIB Software Library and mapped onto CT images aligned to the standard brain space [15]. CST-LL was determined by identifying overlapping voxels between the CST and the lesion mask. The total volume of overlapping CST-LL voxels (in milliliters) was calculated for each patient (see Figure 1) [8,12].

Clinical assessments

Motor impairment severity at admission was assessed using the Brunnstrom Recovery Stage (BRS) [16], which evaluates upper limb function (proximal: shoulder, elbow, forearm; distal: hand, fingers) and lower limb function on the affected side. Each component is rated on a six-point scale (1-6). The total BRS score (BRS total) was calculated by summing the individual component scores, providing an overall measure of hemiparesis severity [17].

To evaluate motor function in both the upper and lower limbs, the Stroke Impairment Assessment Set motor subscale (SIAS-motor) was used [18]. This assessment consists of five domains (arm, finger, hip, knee, and ankle function), each rated on a six-point scale (0-5). The total SIAS-motor score was calculated following the methodology established in prior studies [8].

Additionally, the motor component of the Functional Independence Measure (FIM-motor) was recorded for each patient [19]. This score, ranging from 13 to 91, reflects the patient’s degree of dependence or independence. SIAS-motor and FIM-motor assessments were conducted biweekly, with final scores documented at discharge from the rehabilitation facility. The total length of stay (LOS), encompassing both acute care and rehabilitation periods, was also recorded.

Statistical analysis

Pearson’s correlation analyses were performed to explore relationships among CST-LL, BRS total at admission, and SIAS-motor total at discharge. Additionally, a multivariate regression analysis was conducted to assess the predictive value of CST-LL and BRS total at admission for motor recovery outcomes. The regression model included CST-LL and BRS total as independent variables and SIAS-motor total as the dependent variable. Statistical analyses were performed using the JMP software package (SAS Institute Inc., Cary, North Carolina). A p-value of <0.05 was considered statistically significant.

## Results

**Figure 2:**
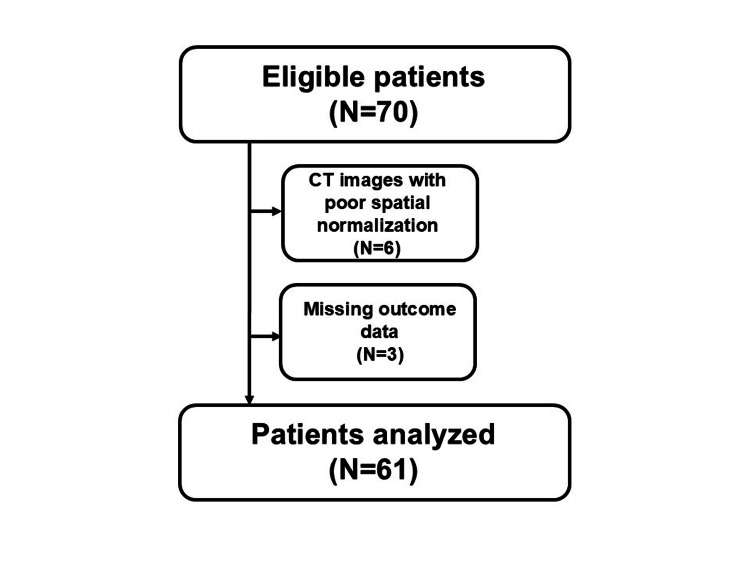
Flow chart of the screening process. CT, computed tomography.

The procedure used to screen patients for the final analytical database is shown in Figure 2. During the study period, 70 patients met the inclusion criteria. After verifying spatial normalization accuracy through visual inspection, six patients were excluded due to the presence of a midline shift. Additionally, three patients were excluded due to incomplete SIAS-motor data. Consequently, 61 patients were included in the final analysis.

Table 1 presents the demographic and clinical characteristics of the study participants. The median age was 66 years (IQR 57-74.5). Of the participants, 35 were male and 26 were female. Lesions were located in the right hemisphere in 35 cases and in the left hemisphere in 26 cases. The median CST-LL was 1.974 mL (IQR 1.113-3.311 mL). Motor impairment was assessed at admission using the BRS. The median BRS scores were 2 (IQR 1-4) for the shoulder/elbow/forearm, 2 (IQR 1-4) for the hand/fingers, and 3 (IQR 2-4.5) for the lower extremities. The total BRS score had a median of 8 (IQR 4-13). Motor function outcomes were evaluated using the SIAS-motor. The median SIAS-motor scores were as follows: arm, 4 (IQR 2-5); finger, 4 (IQR 1-5); hip, 4 (IQR 3-5); knee, 4 (IQR 3-5); and ankle, 4 (IQR 1.5-5). The total SIAS-motor score had a median of 20 (IQR 9.5-24.5). The median score for the Functional Independence Measure motor subscale (FIM-motor) was 81 (IQR 67.5-89). The median LOS was 108 days (IQR 73-152.5).

**Table 1:** Patient demographics and clinical characteristics (N=61). BRS, Brunnstrom recovery stage; FIM-motor, motor component of the Functional Independence Measure; IQR, interquartile range; LOS, total number of hospitalization days including acute care; SIAS-motor, motor component of the Stroke Impairment Assessment Set.

Demographic backgrounds	
Age (years)	Median 66 (IQR 57, 74.5; Range 44-89)
Gender (male/female)	35/26
Lesioned hemisphere (left/right)	26/35
CST-LL (mL)	1.974 (IQR 1.113, 3.311; Range 0.002-7.595)
Clinical severity at admission	
BRS shoulder/elbow/forearm	Median 2 (IQR 1, 4; Range 1-6)
BRS hand/finger	Median 2 (IQR 1, 4; Range 1-6)
BRS lower extremity	Median 3 (IQR 2, 4.5; Range 1-6)
BRS total	Median 8 (IQR 4, 13; Range 3-18)
Outcome assessment	
SIAS-motor arm	Median 4 (IQR 2-5; Range 0-5)
SIAS-motor finger	Median 4 (IQR 1, 5; Range 0-5)
SIAS-motor hip	Median 4 (IQR 3, 5; Range 0-5)
SIAS-motor knee	Median 4 (IQR 3, 5; Range 0-5)
SIAS-motor ankle	Median 4 (IQR 1.5, 5; Range 0-5)
SIAS-motor total	Median 20 (IQR 9.5, 24.5; Range 0-25)
FIM-motor	Median 81 (IQR 67.5, 89; Range 15-91)
LOS	Median 108 (IQR 73, 152.5; Range, 31 - 257)

Table 2 presents the results of the correlation analyses. Significant and strong correlations were observed among CST-LL, BRS total at admission to the acute care hospital, and SIAS-motor total at discharge from the long-term rehabilitation center, with absolute correlation coefficients close to 0.7.

**Table 2:** Results from correlation analyses. BRS, Brunnstrom recovery stage; CST-LL, corticospinal tract lesion load, SIAS-motor, motor component of the Stroke Impairment Assessment Set.

Variables	BRS total	SIAS-motor total
CST-LL	R=-0.697 (P<0.001)	R=-0.668 (P<0.001)
BRS total	-	R=0.745 (P<0.001)

Table 3 summarizes the findings of the multivariate regression analysis. Both CST-LL and BRS total scores at admission were identified as significant predictors of SIAS-motor total scores. The estimated t-values were 4.79 for BRS total and −3.29 for CST-LL, suggesting that these factors had a comparable influence on motor function outcomes. The regression model explained 60.4% of the variance in SIAS-motor total scores.

**Table 3:** Results of multivariate regression analysis. P < 0.05 was considered statistically significant. BRS, Brunnstrom Recovery Stage; CST-LL, corticospinal tract lesion load; SE, standard error; SIAS-motor, motor component of the Stroke Impairment Assessment Set.

Explanatory variable	SIAS-motor total		t	P	
	Estimate	S.E.			
CST-LL	-1.79	0.54	-3.29	0.002	
BRS total	0.82	0.17	4.79	<0.001	
Intercept	13.67	2.51	5.43	<0.001	
Adjusted R^2^	0.604				<0.001

## Discussion

This study identified a novel approach for predicting motor outcomes in patients with intracerebral hemorrhage by integrating clinical severity assessments conducted shortly after stroke onset with CST-LL evaluation using routine CT scans. The findings suggest that combining these two factors could enhance prognostic accuracy and support personalized rehabilitation strategies for stroke recovery.

The correlation analysis results (Table 2) provide valuable insights into the relationship between CST-LL and hemiparesis severity both during the acute phase and in the long term. A strong correlation was observed between CST-LL and hemiparesis severity in the early phase, suggesting a meaningful association between these variables [5,7,8,12]. Moreover, when CST-LL and acute-phase clinical severity were analyzed together, they explained a significant proportion of the variance in long-term hemiparesis severity. Multivariate regression analysis further confirmed that both CST-LL and acute-phase severity independently contributed to stroke prognosis (Table 3).

Previous research has established the clinical relevance of CST-LL, beginning with Zhu et al., who examined T1-weighted images in patients with chronic stroke [20]. Feng et al. later extended this concept by analyzing diffusion-weighted imaging in the acute stroke phase [5]. While diffusion-weighted imaging is primarily utilized for diagnosing acute ischemic stroke, both studies focused on ischemic stroke populations [5,20]. To expand CST-LL’s applicability, multiple research groups have explored its utility in hemorrhagic stroke cases using CT imaging [7,8,12]. Their findings align with those from ischemic stroke cohorts, reinforcing the validity of CST-LL as a prognostic marker across stroke subtypes [5,20]. In our prior work, we confirmed the reproducibility of CST-LL measurements across different datasets, demonstrating methodological consistency [8,12]. While external validation remains necessary, the methodology underlying CST-LL assessment holds promise for clinical implementation.

Recent literature has also emphasized the prognostic significance of initial clinical severity in stroke recovery. For example, the Shoulder Abduction, Finger Extension (SAFE) score is a well-recognized tool for predicting upper limb function recovery [21]. This score has been strongly linked to upper extremity outcomes and is widely used in clinical practice [22]. Assessing acute-phase hemiparesis severity allows clinicians to tailor rehabilitation interventions and set realistic recovery goals based on individual patient needs. The severity of initial impairment serves as a key prognostic indicator, helping guide rehabilitation strategies and improve patient outcomes.

In this study, we selected specific scoring systems for evaluating acute-phase hemiparesis severity and long-term motor outcomes based on their clinical utility. The BRS was used during acute care due to its straightforward application [16]. However, because the BRS provides a generalized assessment of lower limb function, the Stroke Impairment Assessment Set (SIAS-motor) was employed for long-term evaluation, as it allows for a more detailed assessment of hip, knee, and ankle function [18]. Given the critical role of regaining ambulation for functional independence in chronic stroke patients, a more granular evaluation of lower extremity function is advantageous [23]. To assess the comparability of these two scoring systems, we preliminarily examined their correlation in a subset of patients who were evaluated using both scales during long-term rehabilitation. The Spearman’s correlation coefficient for the BRS and SIAS-motor total scores at discharge was 0.98 (N=15), demonstrating a strong association between these measures.

Previous literature has indicated that injury to the CST is more closely associated with the severity of upper extremity impairment than with that of lower extremity impairment [1-4]. One possible explanation lies in neuroanatomical differences within the CST related to the control of upper and lower extremities. The CST traverses relatively small and compact regions, including the posterior limb of the internal capsule and the cerebral peduncle. The posterior limb of the internal capsule lies adjacent to the thalamus and putamen, which are common sites of origin for intracerebral hemorrhage. Traditionally, the motor somatotopy in this region has been thought to follow an anteromedial pattern for the upper extremities and a posterolateral pattern for the lower extremities. However, recent studies using diffusion tensor imaging and functional magnetic-resonance imaging have demonstrated a different organization, with the upper extremity represented anterolaterally and the lower extremity posteromedially, along with considerable inter-individual variability in this pattern [24,25]. Accordingly, motor somatotopy is now regarded as more complex than previously believed. In this line, in the present study, we did not differentiate the CST injury according to upper or lower extremity pathways.

This study has several limitations. First, this study included only patients with hemorrhages localized to the thalamus and/or putamen, which may restrict the generalizability of our findings. Future research should consider hemorrhages in other brain regions to assess broader applicability. Second, only functionally independent patients experiencing their first-ever stroke were included, limiting the extension of our results to individuals who required assistance before their stroke. Further studies are needed to validate these findings in a more diverse patient population. Third, our sample size was relatively small (N=61), which may impact the statistical power and generalizability of the results. While small sample sizes are common in neuroimaging research, larger studies would strengthen the reliability of these findings. Despite these constraints, our study provides valuable insights and highlights the need for further investigations to address these limitations and improve understanding across different patient populations and lesion locations.

## Conclusions

This study underscores the importance of combining acute-phase clinical severity, assessed by the BRS, with CST-LL measured using routine CT imaging to predict motor recovery in patients with intracerebral hemorrhage. Our findings indicate that these two factors serve as independent yet complementary predictors, together explaining a substantial portion of motor outcome variability. By incorporating both clinical severity and CST-LL into early assessments, clinicians can refine prognosis predictions, tailor rehabilitation strategies, and ultimately enhance patient recovery in stroke rehabilitation.

## References

[1] Stinear CM, Smith MC, Byblow WD (2019). Prediction tools for stroke rehabilitation. Stroke.

[2] Stinear CM (2017). Prediction of motor recovery after stroke: advances in biomarkers. Lancet Neurol.

[3] Boyd LA, Hayward KS, Ward NS (2017). Biomarkers of stroke recovery: consensus-based core recommendations from the Stroke Recovery and Rehabilitation Roundtable. Neurorehabil Neural Repair.

[4] Rosso C, Lamy JC (2020). Prediction of motor recovery after stroke: being pragmatic or innovative?. Curr Opin Neurol.

[5] Feng W, Wang J, Chhatbar PY (2015). Corticospinal tract lesion load: an imaging biomarker for stroke motor outcomes. Ann Neurol.

[6] Ito KL, Kim B, Liu J (2022). Corticospinal tract lesion load originating from both ventral premotor and primary motor cortices are associated with post-stroke motor severity. Neurorehabil Neural Repair.

[7] Lam TK, Cheung DK, Climans SA (2020). Determining corticospinal tract injury from stroke using computed tomography. Can J Neurol Sci.

[8] Uchiyama Y, Domen K, Koyama T (2021). Outcome prediction of patients with intracerebral hemorrhage by measurement of lesion volume in the corticospinal tract on computed tomography. Prog Rehabil Med.

[9] Heo J, Yoon JG, Park H, Kim YD, Nam HS, Heo JH (2019). Machine learning-based model for prediction of outcomes in acute stroke. Stroke.

[10] Wang W, Kiik M, Peek N (2020). A systematic review of machine learning models for predicting outcomes of stroke with structured data. PLoS One.

[11] Christidi F, Orgianelis I, Merkouris E (2024). A comprehensive review on the role of resting-state functional magnetic resonance imaging in predicting post-stroke motor and sensory outcomes. Neurol Int.

[12] Koyama T, Mochizuki M, Uchiyama Y, Domen K (2024). Outcome prediction by combining corticospinal tract lesion load with diffusion-tensor fractional anisotropy in patients after hemorrhagic stroke. Prog Rehabil Med.

[13] Miyamoto S, Ogasawara K, Kuroda S (2022). Japan Stroke Society guideline 2021 for the treatment of stroke. Int J Stroke.

[14] Jenkinson M, Beckmann CF, Behrens TE, Woolrich MW, Smith SM (2012). FSL. Neuroimage.

[15] Hua K, Zhang J, Wakana S (2008). Tract probability maps in stereotaxic spaces: analyses of white matter anatomy and tract-specific quantification. Neuroimage.

[16] Brunnstrom S (1966). Motor testing procedures in hemiplegia: based on sequential recovery stages. Phys Ther.

[17] Uchiyama Y, Domen K, Koyama T (2023). Brain regions associated with Brunnstrom and functional independence measure scores in patients after a stroke: a tract-based spatial statistics study. J Phys Ther Sci.

[18] Tsuji T, Liu M, Sonoda S, Domen K, Chino N (2000). The stroke impairment assessment set: its internal consistency and predictive validity. Arch Phys Med Rehabil.

[19] Heinemann AW, Linacre JM, Wright BD, Hamilton BB, Granger C (1993). Relationships between impairment and physical disability as measured by the functional independence measure. Arch Phys Med Rehabil.

[20] Zhu LL, Lindenberg R, Alexander MP, Schlaug G (2010). Lesion load of the corticospinal tract predicts motor impairment in chronic stroke. Stroke.

[21] Nijland RH, van Wegen EE, Harmeling-van der Wel BC, Kwakkel G (2010). Presence of finger extension and shoulder abduction within 72 hours after stroke predicts functional recovery: early prediction of functional outcome after stroke: the EPOS cohort study. Stroke.

[22] Stinear CM, Byblow WD, Ackerley SJ, Smith MC, Borges VM, Barber PA (2017). PREP2: a biomarker-based algorithm for predicting upper limb function after stroke. Ann Clin Transl Neurol.

[23] Maeshima S, Osawa A, Nishio D, Hirano Y, Kigawa H, Takeda H (2013). Diffusion tensor MR imaging of the pyramidal tract can predict the need for orthosis in hemiplegic patients with hemorrhagic stroke. Neurol Sci.

[24] Holodny AI, Gor DM, Watts R, Gutin PH, Ulug AM (2005). Diffusion-tensor MR tractography of somatotopic organization of corticospinal tracts in the internal capsule: initial anatomic results in contradistinction to prior reports. Radiology.

[25] Ino T, Nakai R, Azuma T, Yamamoto T, Tsutsumi S, Fukuyama H (2007). Somatotopy of corticospinal tract in the internal capsule shown by functional MRI and diffusion tensor images. Neuroreport.

